# Efficacy and safety of Lingbao Huxin Dan in the treatment of sick sinus syndrome: a randomized, single-blind, placebo-controlled, crossover trial

**DOI:** 10.3389/fcvm.2026.1821435

**Published:** 2026-08-12

**Authors:** Yue Wu, Yucheng Wang, Xing Zhou, Zhijun Wang, Dongming Lin, Shumin Lv, Xinbin Zhou, Xinxin Shi, Liming He, Yaping Zhou, Shuwei Huang

**Affiliations:** Department of Cardiovascular Medicine, The First Affiliated Hospital of Zhejiang Chinese Medical University (Zhejiang Provincial Hospital of Chinese Medicine), Hangzhou, Zhejiang, China

**Keywords:** atrial pacing percentage, crossover study, heart rate variability, Lingbao Huxin Dan, pacemaker, sick sinus syndrome

## Abstract

**Background:**

Permanent pacemaker implantation is the standard treatment for symptomatic sick sinus syndrome (SSS), but it does not reverse the underlying sinus node dysfunction. Lingbao Huxin Dan (LBHXD) may benefit patients with bradyarrhythmia. However, robust clinical evidence regarding its efficacy and safety in patients with SSS, particularly those with pacemakers, is lacking.

**Methods:**

A randomized, crossover trial was conducted. Thirty patients with SSS and implanted DDD pacemakers were enrolled and randomly assigned to one of two treatment sequences: LBHXD followed by placebo, or placebo followed by LBHXD. Each treatment period lasted 4 weeks, separated by a 2-week washout interval.

**Results:**

25 patients completed the trial. Following propensity score matching, 17 patients were included in the final analysis. Compared to the placebo phase, LBHXD administration resulted in a significantly greater reduction in AP% (38.99% ± 20.67% vs. 48.03% ± 21.95%, *P* < 0.01). LBHXD also significantly increased several HRV indices, including SDNN, SDANN, rMSSD, and SDNN index (all *P* < 0.05), and elevated the mean and maximum HR (*P* < 0.05). No significant changes were observed in ventricular pacing percentage, left atrial diameter, or left ventricular ejection fraction.

**Conclusion:**

Adjunctive therapy with Lingbao Huxin Dan can effectively reduce atrial pacing dependency, improve heart rate variability, and increase heart rate in patients with SSS and pacemakers, demonstrating a favorable safety profile. These findings suggest that LBHXD may enhance intrinsic sinoatrial node function and autonomic regulation, supporting its potential role as a complementary therapy in an integrative medicine approach to managing SSS.

**Clinical Trial Registration:**

https://www.chictr.org.cn/showproj.html?proj=187346, Chinese Clinical Trial Registry ChiCTR2300067497.

## 1 Introduction

Sick sinus syndrome (SSS) is a spectrum of clinical disorders arising from structural or functional abnormalities in the sinoatrial node, leading to impaired pacemaker activity and disrupted impulse conduction. These pathophysiological disturbances compromise normal cardiac rhythm regulation and result in a range of arrhythmias, including sinus bradycardia, sinus arrest, and sinoatrial block. A hallmark feature of SSS is the alternation between bradyarrhythmias and tachyarrhythmias—commonly referred to as tachycardia-bradycardia syndrome—which contributes to inadequate chronotropic response during physical activity. This dysregulation manifests clinically as dizziness, presyncope, syncope, and palpitations (1). Epidemiological data indicate that the prevalence of SSS is approximately 0.8 per 1,000 individuals (2). Although the incidence increases markedly with age, making advanced age the most prominent risk factor, SSS can occur across all adult age groups (3). Notably, nearly 50% of patients with SSS develop the tachycardia-bradycardia variant, which is independently associated with an elevated risk of thromboembolic events, particularly stroke, as well as increased all-cause mortality (4). With the ongoing global demographic shift toward an aging population, the burden of SSS is expected to rise substantially over the coming decades (5).

At present, permanent pacemaker implantation remains the standard of care for symptomatic SSS, offering significant improvements in symptom control and quality of life. However, this intervention does not address the underlying disease progression, necessitates long-term follow-up, and has not been definitively shown to reduce overall mortality. Moreover, device implantation carries inherent risks, including procedural complications such as infection, lead dislodgement, and pneumothorax, as well as long-term device-related morbidities (6, 7). Pharmacological management of SSS remains limited. No drug is currently approved for long-term correction of sinus node dysfunction, largely due to insufficient efficacy and unfavorable safety profiles. In acute settings, agents such as atropine or epinephrine may transiently increase heart rate (HR) but are unsuitable for chronic use owing to their adverse effect burden (7). While phosphodiesterase inhibitors like theophylline exhibit positive chronotropic effects in some patients with sinus node dysfunction, there is no robust evidence supporting their ability to modify disease course or improve long-term outcomes (8). The rising prevalence of SSS, coupled with increasing demand for device-based therapies, imposes a growing strain on healthcare systems, reflected in higher rates of hospitalization and escalating medical costs (9). Given these challenges, there is a compelling need to explore adjunctive or alternative therapeutic strategies, particularly for patients managed conservatively or those with residual symptoms despite pacing.

In Traditional Chinese Medicine (TCM), SSS is categorized under syndromes such as “slow pulse disorder” and “palpitations”, with pathogenesis primarily attributed to “deficiency of Yang Qi, blood stasis, and cold coagulation.” Therapeutic principles emphasize “replenishing Qi, warming Yang, promoting blood circulation, and resolving stasis” to restore cardiovascular homeostasis. Lingbao Huxin Dan (LBHXD) is a patented Chinese herbal formulation composed of nine ingredients (Table 1): musk (*Moschus*, Shexiang), dried toads venom (*Bufonis Venenum*, Chansu), bezoar (*Bovis Calculus*, Niuhuang), synthetic borneol (*Borneolum Syntheticum*, Bingpian), red ginseng (*Ginseng Radix et Rhizoma Rubra*, Hongshen), Sanchi (*Notoginseng Radix et Rhizoma*, Sanqi), amber (*Succinum*, Hupo), danshen (*Salvia miltiorrhiza Bunge*, Danshen), and storax (*Liquidambar orientalis Mill*, Suhexiang) [Approval No. Su Wei Yao Zhun Zi (85) 1770–1]. It has been used clinically in China for many years and is indicated for conditions involving Qi deficiency and blood stasis, with documented effects in improving microcirculation, enhancing myocardial perfusion, and modulating autonomic tone (10). Emerging evidence suggests beneficial effects of LBHXD in managing bradyarrhythmias, angina pectoris, and ischemic heart disease, with reported alleviation of symptoms including chest tightness, chest pain, and palpitations (11, 12). Despite these promising observations, high-quality clinical data on the efficacy and safety of LBHXD specifically in patients with SSS—particularly those with implanted pacemakers—are scarce. Therefore, this study aims to evaluate the potential of LBHXD as an adjunctive therapy in improving arrhythmia burden and symptom control among SSS patients post-pacemaker implantation, while rigorously assessing its safety profile. The results may offer preliminary insights into the potential pharmacodynamic mechanisms of this herbal formulation and support the integration of evidence-based TCM approaches into comprehensive management strategies for SSS. The plant name has been checked with Chinese Pharmacopoeia (2020) and MPNS (http://mpns.kew.org).

**Table 1:** Composition of Lingbao Huxin Dan prescription.

Chinese medicine name	Latin name	Scientific name	Family	Medicinal part	Ratio
Shexiang	*Moschus*	*Moschus spp.*	Cervidae	Dried secretion from the preputial gland of mature male deer (Natural Source)	0.3%
Chansu	*Bufonis Venenum*	*Papaver somniferum L.*	Bufonidae	Dried parotid and skin gland secretions	3.1%
Niuhuang	*Bovis Calculus*	*Panax ginseng C.A.Mey.*	Bovidae	Biliary calculus (Natural Source)	11.0%
Bingpian	*Borneolum Syntheticum*	*Dryobalanops aromatica C.F.Gaertn.*	Synthetic (derived from turpentine oil or camphor)	Synthetic crystals	3.5%
Hongshen	*Ginseng Radix et Rhizoma Rubra*	*Panax ginseng C.A.Mey.*	Araliaceae	Dried root and rhizome	17.6%
Sanqi	*Notoginseng Radix et Rhizoma*	*Basella alba L.*	Araliaceae	Dried root and rhizome	17.6%
Hupo	*Succinum*	*Magnolia officinalis Rehder & E.H.Wilson*	Pinaceae	fossilized resin	8.8%
Danshen	*Sal*via *miltiorrhiza Bunge*	*Sal*via *miltiorrhiza Bunge*	Lamiaceae	Dried root and rhizome	29.3%
Suhexiang	*Liquidambar orientalis Mill*	*Liquidambar orientalis Mill.*	Hamamelidaceae	Oleoresin exudate	8.7%

## 2 Materials and methods

### 2.1 Study design

This study employed a randomized, single-blind, self-controlled crossover design to evaluate the efficacy of LBHXD in patients with SSS. Participants were recruited from the First Affiliated Hospital of Zhejiang Chinese Medical University between April 2023 and June 2025. The study protocol was approved by the Ethics Committee of the First Affiliated Hospital of Zhejiang Chinese Medical University (Approval No.: 2022-KL-090-01) and registered at the Chinese Clinical Trial Registry (ChiCTR2300067497). Written informed consent was obtained from all participants or their legally authorized representatives prior to enrollment, in accordance with the principles of the Declaration of Helsinki.

### 2.2 Diagnostic criteria

SSS was diagnosed based on established clinical and electrophysiological criteria, excluding confounding factors such as neuroendocrine dysregulation or medication-induced bradycardia. Diagnosis required fulfillment of at least one of the following criteria, with all diagnoses confirmed by correlation of electrocardiographic findings with clinical symptoms (e.g., dizziness, presyncope, syncope, or palpitations) (2): (1) persistent sinus bradycardia with HR <50 bpm; (2) second-degree type II sinoatrial block; (3) sinus pauses exceeding 2 s (a screening threshold widely used in Chinese clinical practice (13); however, all enrolled patients in this study met the more stringent criterion of pauses >3 s with symptom correlation, consistent with the 2018 ACC/AHA/HRS Guideline definition (2); (4) coexistence of atrioventricular block and sinoatrial block; (5) slow-fast syndrome, characterized by alternating episodes of bradyarrhythmia and tachyarrhythmia; (6) 24 h dynamic electrocardiography (DCG) showing total cardiac beats ≤80,000, with mean, minimum, and maximum HRs ≤55, ≤40, and ≤90 bpm, respectively, with or without associated arrhythmias; (7) post-atropine test demonstrating a maximal sinus rate <90 bpm or emergence of sinoatrial block, sinus arrest, junctional rhythm, or atrial fibrillation; (8) electrophysiological testing revealing corrected sinus node recovery time >550 ms, sinus node recovery time >1,500 ms, effective refractory period >600 ms, or conduction time >120 ms.

### 2.3 Inclusion and exclusion criteria

Inclusion criteria were as follows: (1) age 18–80 years, regardless of sex; (2) implanted DDD-mode pacemaker with diagnostic recording capability; (3) no cardiac surgery within the preceding six months; (4) absence of severe heart failure (NYHA class III–IV); (5) TCM diagnosis of Qi deficiency and blood stasis syndrome; (6) provision of informed consent and ability to comply with study procedures. In cases where both participant and legal guardian were unable to read or comprehend the consent form, a third-party witness co-signed the document.

Exclusion criteria included: (1) significant comorbidities involving hepatic, renal, hematologic, or central nervous systems, or active psychiatric disorders; (2) hepatic dysfunction defined as alanine aminotransferase or aspartate aminotransferase >2 × upper limit of normal (ULN), or total bilirubin > ULN; (3) renal impairment with serum creatinine >1.2 × ULN or creatinine clearance (CrCl) < 50 mL/min (calculated via Cockcroft-Gault formula); (4) pregnancy, lactation, or intention to conceive during the study period without effective contraception; (5) participation in another interventional clinical trial within 3 months prior to screening; (6) any condition deemed by the investigator to compromise compliance or safety; (7) known hypersensitivity to any component of the investigational product.

### 2.4 Randomization and blinding

**Figure 1:**
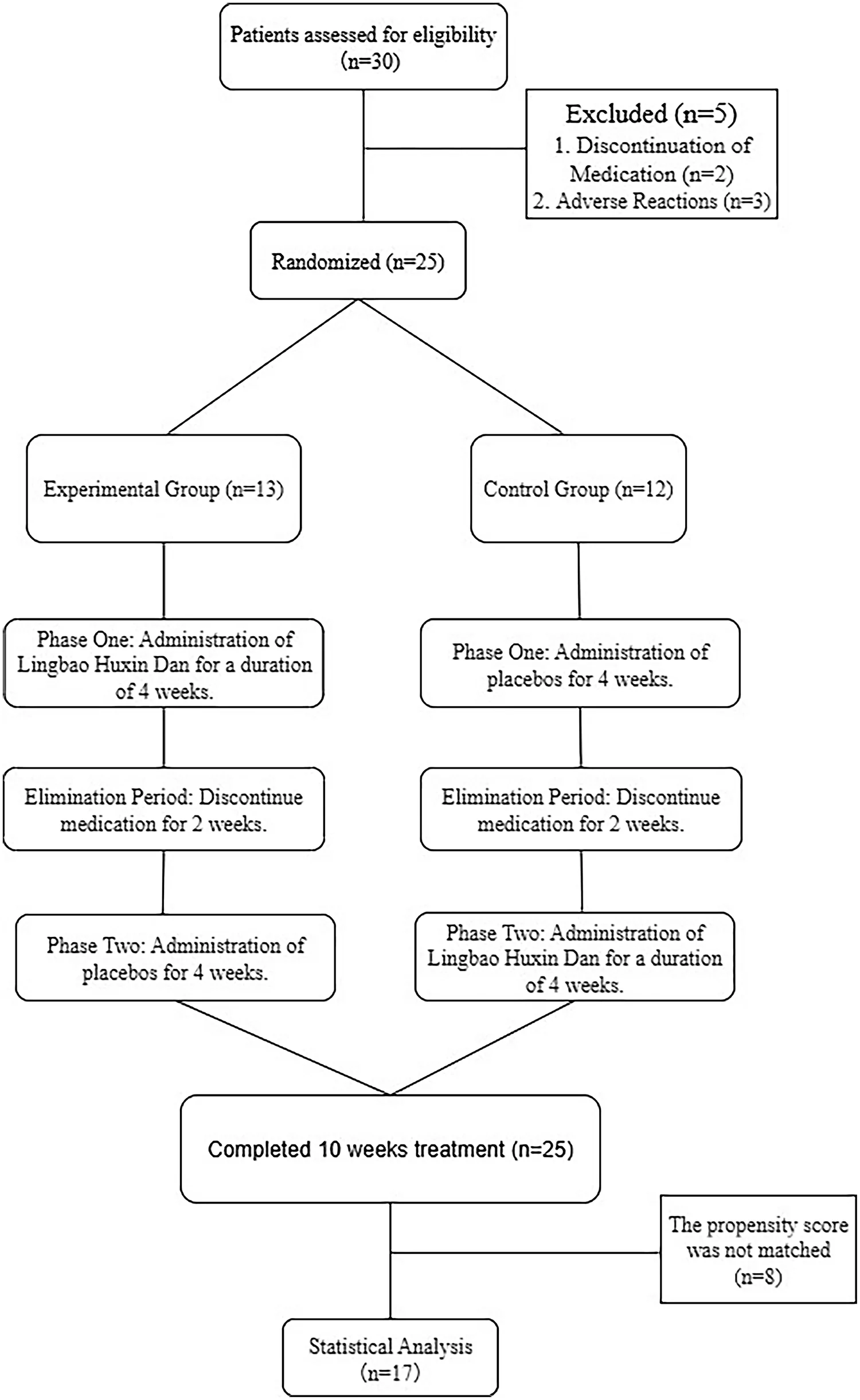
Enrollment and randomization of participants in the study. The arrows in the figure show how the subjects were included, excluded, randomized and followed up.

Randomization was performed using SPSS 26.0 software to generate a simple random allocation sequence. Participants were assigned in a 1:1 ratio to one of two treatment sequences. The study utilized a single-blind design, with participants blinded to treatment assignment. Both active drug and placebo were identically packaged in terms of appearance, color, taste, and labeling. Allocation codes were managed centrally, and participants received only their subject ID and medication code, ensuring maintenance of blinding throughout the trial (Figure 1).

To further reduce potential bias, we implemented the following additional measures: (1) centralized management of randomization coding, with participants only aware of their subject number and drug code; (2) standardized assessment of all outcome measures at every study visit; and (3) blinded data analysis conducted by personnel outside the research team who were unaware of treatment assignments. These measures collectively reduced the risk of detection and assessment bias.

### 2.5 Preparation of LBHXD and placebo

LBHXD were manufactured by Leiyunshang Pharmaceutical Group Co., Ltd. (National Drug Approval No.: Z32021181), with a formulation of 0.08 g per 10 pills, packaged as 12 pills × 2 bottles/box. The placebo, composed primarily of starch and other inert excipients, was supplied by the same manufacturer and matched the active product in dosage form, packaging, and sensory characteristics (0.08 g per 10 pills, 12 pills × 2 bottles/box).

All pharmaceuticals used in this study were manufactured by certified pharmaceutical companies in strict accordance with national standards and Good Manufacturing Practice regulations for pharmaceutical production. LBHXD and the placebo were produced by Leiyunshang Pharmaceutical Group Co., Ltd. (National Drug Approval No. Z32021181). The formulation is 0.08 g per 10 pills, with each box containing two bottles of 12 pills. The placebo for Lingbao Huxin Dan, provided by the same manufacturer, consists primarily of starch and other inert excipients, matching LBHXD in appearance, packaging, odor, and dosage form.

**Figure 2:**
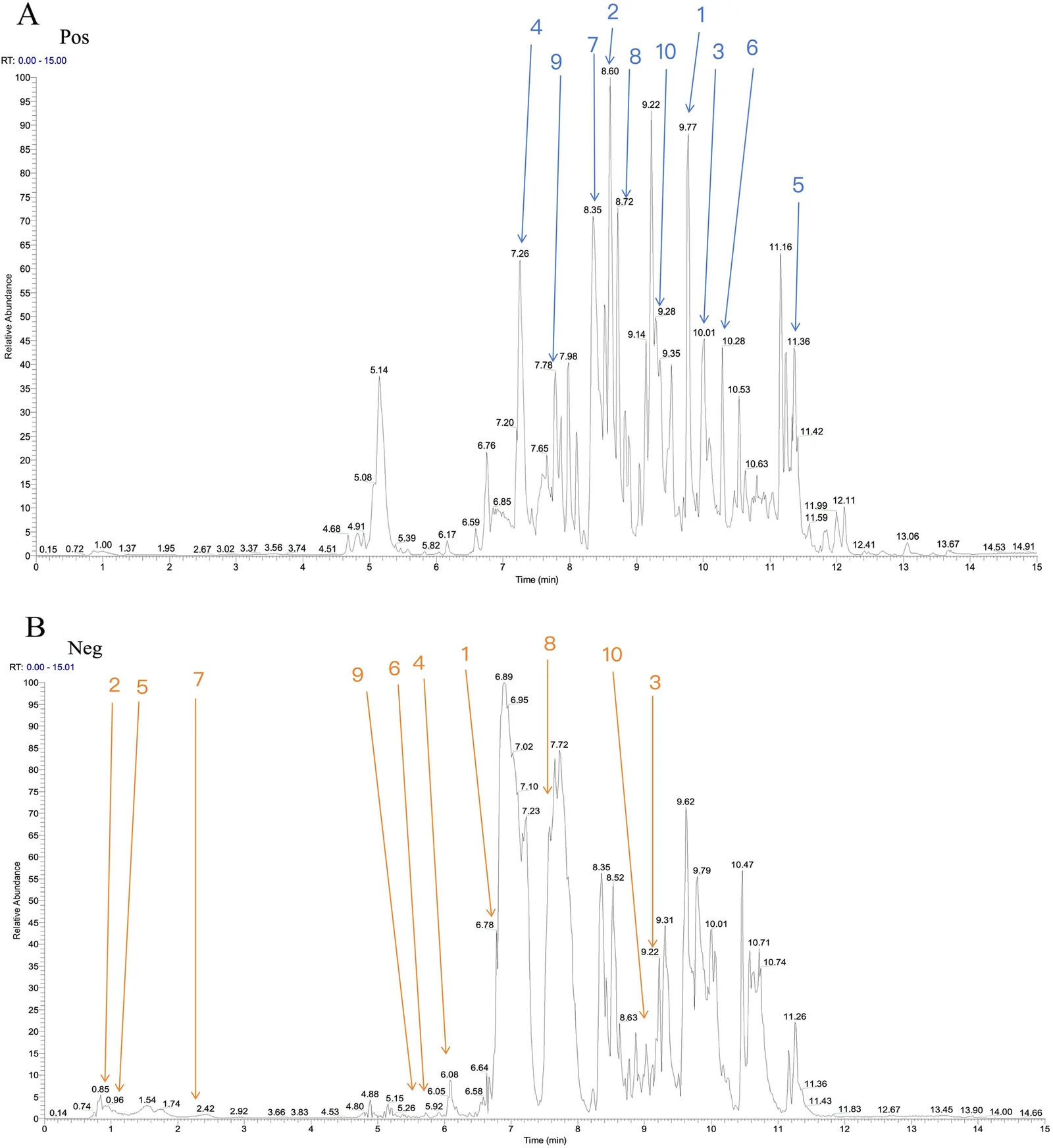
UPLC-Q-MS mass spectrograms of extraction of LBHXD. **(A)** Positive pattern. **(B)** Negative pattern.

The quality of LBHXD was rigorously assessed by the manufacturer through a comprehensive battery of tests, including microscopic and physicochemical identification, thin-layer chromatography, moisture content analysis, disintegration time testing, and microbial limit evaluation. All results conformed to both industry standards and the specifications outlined in the Chinese Pharmacopoeia. The assay of marker compounds fulfilled regulatory requirements, and microbiological testing confirmed the absence of *Escherichia coli* and *Salmonella spp.*; levels of molds, yeasts, aerobic bacteria, and Gram-negative bacteria were within acceptable limits. Chemical profiling of LBHXD was performed using ultra-high performance liquid chromatography coupled with quadrupole time-of-flight mass spectrometry (UPLC-QTOF-MS). By comparison with commercially available reference standards, a total of 222 constituents were tentatively identified. The ten most abundant compounds, ranked by ion intensity in both positive and negative ionization modes, are summarized in the Supplementary Material and Figure 2. All products were stored under cool, dry, and well-ventilated conditions prior to use. Full quality control documentation for both LBHXD and the placebo is included in the Supplementary Material.

### 2.6 Treatment protocols and group allocation

This study employed a randomized, placebo-controlled crossover design, consisting of two 4-week intervention periods separated by a 2-week washout phase. During the screening period, eligible participants underwent baseline assessments and pacemaker optimization, including setting the base pacing rate to 60 bpm and deactivating the rate-responsive function to ensure consistent cardiac pacing conditions throughout the study.

Participants were then randomly assigned to one of two sequences: experimental Group received LBHXD for the first 4 weeks, followed by 2 weeks off-treatment, then placebo for the subsequent 4 weeks; Control Group received placebo first, followed by the active treatment after the washout. Assessments were conducted at baseline (Day 1), Week 4, and Week 10, with a ± 3-day window allowed for visit scheduling.

The total study duration was 10 weeks, with scheduled visits at baseline (enrollment), Week 4 (end of first intervention), and Week 10 (end of second intervention). At each visit, participants returned to the clinical site for standardized evaluations and safety monitoring.

### 2.7 Efficacy assessments

The primary efficacy endpoint was the atrial pacing (AP) proportion, assessed using dual data sources: device-based telemetry from pacemaker programming logs and DCG. This dual-source approach enhanced measurement reliability and minimized potential bias in pacing assessment.

Secondary efficacy endpoints included: A. Time-domain heart rate variability (HRV) parameters derived from DCG recordings: standard deviation of all normal R-R intervals (SDNN), reflecting overall HRV; standard deviation of the average normal R-R intervals across each 5 min segment over 24 h (SDANN), indicating long-term trends in HRV; mean of the standard deviations of normal R-R intervals within each 5 min interval over 24 h (SDNN index); root mean square of successive differences between adjacent normal R-R intervals (RMSSD), representing short-term HRV; and proportion of successive normal R-R intervals differing by more than 50 ms (PNN50), quantifying parasympathetic modulation.

B. DCG-derived metrics: mean, minimum, and maximum HRs during the monitoring period, providing insight into autonomic regulation and circadian rhythm patterns.

C. Echocardiographic measurements: changes in left atrial anteroposterior diameter and left ventricular ejection fraction (LVEF), used to evaluate structural and systolic functional adaptations. All echocardiographic examinations were performed by certified sonographers with ≥5 years of experience in cardiac imaging. Offline analysis of echocardiographic images was conducted by two independent, blinded readers who were unaware of both treatment allocation and assessment time points. This blinded, consensus-based analytical approach minimized detection bias and enhanced measurement reproducibility.

Safety assessments encompassed vital sign monitoring, complete blood count, urinalysis, hepatic and renal function tests, serial 12-lead electrocardiograms, and systematic recording of adverse events throughout the study period.

### 2.8 Statistical analyses

All clinical data were analyzed using SPSS software (version 25.0). To appropriately account for the crossover design, the following analytical procedures were performed. Carryover effects were assessed by comparing pre-treatment baseline values before the first and second intervention periods using independent samples t-tests. Specifically, we compared AP% values measured at the beginning of Period 1 (baseline) and at the beginning of Period 2 (following the 2-week washout period). The primary efficacy analysis was conducted using paired comparisons within each participant: for the primary endpoint (AP%), the difference between LBHXD and placebo phases was analyzed using paired t-tests (normally distributed data) or Wilcoxon signed-rank tests (non-normally distributed data). Secondary endpoints were analyzed using analogous paired methods. Baseline demographic characteristics between the two sequence groups were compared using independent samples t-tests for continuous variables and chi-square tests for categorical variables, as appropriate. Data distribution was evaluated using the Shapiro–Wilk test. Normally distributed continuous variables were expressed as mean ± standard deviation (M ± SD), and non-normally distributed variables were summarized as median (interquartile range). To adjust for potential confounding factors, propensity score matching (PSM) was applied prior to primary analysis, balancing baseline characteristics between groups. All statistical tests were two-sided, and a *P*-value < 0.05 was considered statistically significant.

## 3 Results

### 3.1 Baseline clinical characteristics

A total of 30 eligible participants was enrolled in the study and randomly assigned to either the experimental or control group (*n* = 15 per group) following pacemaker parameter optimization. Five participants were subsequently excluded due to premature discontinuation of treatment or adverse events, leaving 25 participants who completed the full protocol and were included in the final analysis—13 in the experimental group and 12 in the control group.

No significant differences were observed between the two groups in baseline demographic variables, including age, sex, body mass index (BMI), and disease duration (*P* > 0.05). A proportion of participants had one or more concomitant cardiovascular or metabolic comorbidities, such as hypertension, type 2 diabetes mellitus, and coronary artery disease, with a balanced distribution across groups (Table 2).

**Table 2:** Baseline demographic characteristics.

Characteristics	Experimental group (*n* = 13)	Control group (*n* = 12)	*P* value
Gender (Male, %)	8 (61.54)	7 (58.33)	0.317
Age (Years, M ± SD)	66.69 ± 5.12	69.58 ± 5.35	0.392
Medical history (Months, M ± SD)	69.77 ± 52.25	53.39 ± 45.83	0.270
BMI (kg m^2^, M ± SD)	25.31 ± 6.19	25.07 ± 3.04	0.903
Pacemaker Brand	Medtronic (*n* = 6) Abbott (*n* = 4) Biotronik (*n* = 2) Boston Scientific (*n* = 1)	Abbott (*n* = 7) Biotronik (*n* = 3) Medtronic (*n* = 2)	
Comorbidities	Hypertension (*n* = 6) Coronary Heart Disease (*n* = 4) Diabetes (*n* = 2)	Hypertension (*n* = 7) Coronary Heart Disease (*n* = 3) Diabetes (*n* = 1) Stroke(*n* = 1)	

The medical history of the subjects refers to the time from the initial diagnosis of sick sinus syndrome to the enrollment in this study.

Prior to the primary efficacy analysis, the fundamental assumptions of the crossover design were evaluated. Assessment of carryover effects revealed no significant difference in pre-treatment AP% values between the first and second intervention periods (*P* > 0.05), indicating the absence of clinically meaningful residual effects from the first period to the second.

### 3.2 Pacemaker-derived pacing parameters

**Figure 3:**
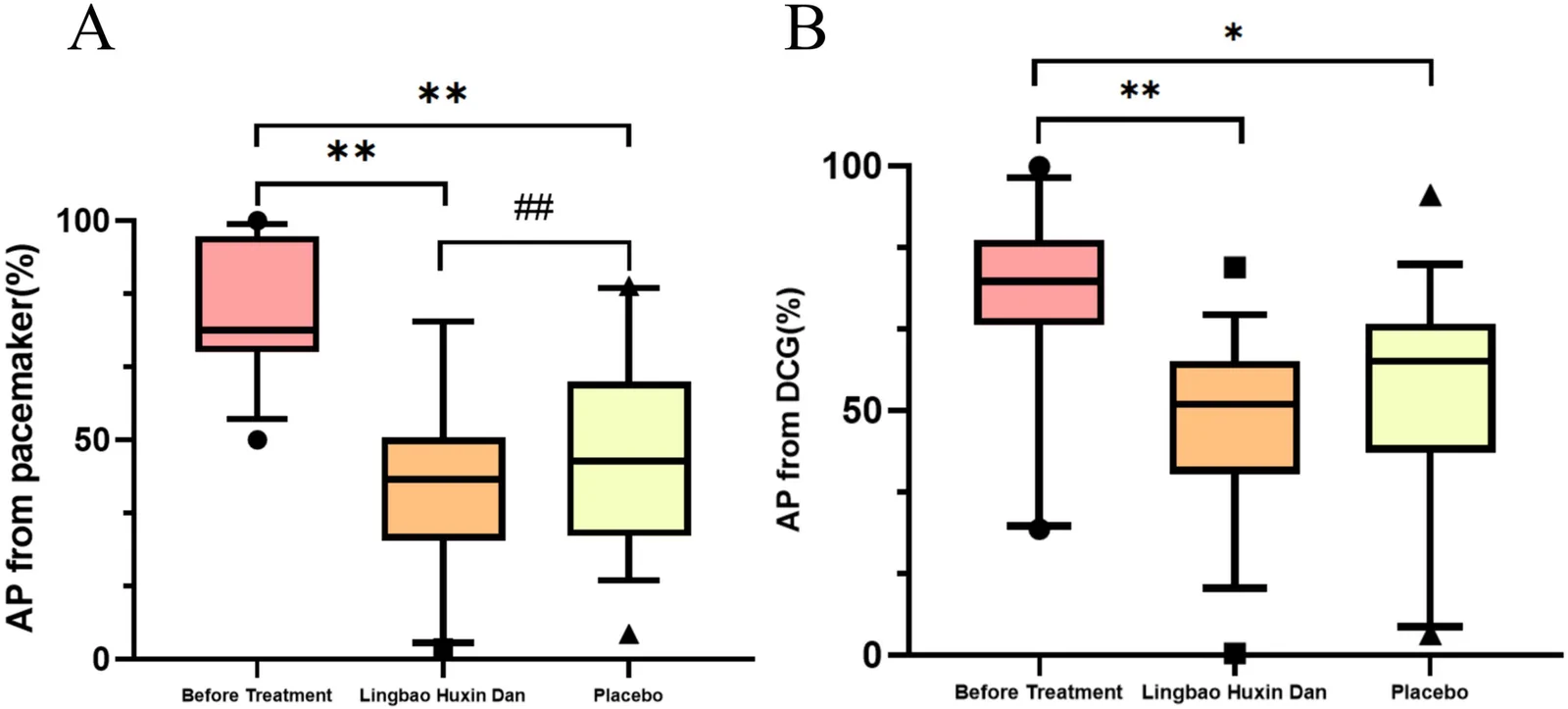
Proportion of AP before and after intervention (%; 10th–90th percentile). **(A)** Displays pacemaker programming parameters, and **(B)** presents the proportion of AP derived from DCG. Compared with the pre-intervention baseline, *P < 0.05 and **P < 0.01; compared with the placebo period, #*P* < 0.05 and ##*P* < 0.01. AP, atrial pacing proportion; DCG, dynamic electrocardiography.

Prior to primary efficacy analysis, propensity score matching was performed with a caliper width of 0.05. Eight participants were identified as unmatched and were excluded from subsequent paired comparisons. As shown in Table 3 and Figure 3, AP proportion significantly decreased during both intervention periods (*P* < 0.05). However, the reduction was significantly greater in the LBHXD period compared to placebo (38.99 ± 20.67% vs. 48.03 ± 21.95%, *P* < 0.01), indicating a greater intrinsic cardiac rhythm preservation with active treatment. No statistically significant changes were observed in ventricular pacing (VP) proportion (*P* > 0.05), suggesting no detrimental impact on device performance.

**Table 3:** Changes in programmed parameters before and after each intervention period.

Parameters	Before treatment (*n* = 17)	Placebo (*n* = 17)	Lingbao Huxin Dan (*n* = 17)
Atrial Pacing Ratio (%, M ± SD)	78.29 ± 15.41^a,b^	48.03 ± 21.95^a,c^	38.99 ± 20.67^b,c^
Ventricular Pacing Ratio (%, p25, p75)	1.05 (0.33，31.65)	1.00 (1.00，6.28)	1.25 (1.00，2.90)
Pacing AV Interval (ms, M ± SD)	236.50 ± 21.44	241.67 ± 12.15	234.5 ± 21.00
Sensing AV interval (ms, M ± SD)	212.50 ± 25.88	222.22 ± 18.63	210.00 ± 25.75
Atrial sensing (mV, M ± SD)	2.72 ± 1.37	3.36 ± 1.52	3.12 ± 1.53
Ventricular Sensing (mV, M ± SD)	8.79 ± 3.29	10.64 ± 3.04	9.64 ± 2.67
Atrial Threshold (V, M ± SD)	0.84 ± 0.45	0.75 ± 0.28	0.67 ± 0.23
Ventricular Threshold (V, M ± SD)	0.85 ± 0.30	1.10 ± 0.84	0.92 ± 0.34
Atrial Impedance (Ω, M ± SD)	534.81 ± 107.54	449.81 ± 89.85	524.44 ± 102.68
Ventricular Impedance (Ω, M ± SD)	614.00 ± 180.00	542.31 ± 116.74	562.50 ± 115.77

Superscript letters denote statistically significant differences: a, compared with placebo group before intervention; b, compared with Lingbao Huxin Dan group before intervention; c, between placebo and Lingbao Huxin Dan groups.

### 3.3 Time-domain HRV

Compared to the placebo phase, administration of LBHXD was associated with significant increases in multiple time-domain HRV indices: SDNN (*P* < 0.05), SDANN (*P* < 0.05), rMSSD (*P* < 0.05), and SDNN index (*P* < 0.05), reflecting enhanced autonomic modulation and improved HR dynamics. When compared to baseline, all four parameters showed marked improvement after the active treatment phase (*P* < 0.05). During the placebo phase, a similar trend was observed, but only SDNN reached statistical significance (*P* < 0.05), suggesting a modest nonspecific effect possibly attributable to study participation or temporal factors (Table 4).

**Table 4:** Changes in time-domain indices of heart rate variability before and after each intervention period.

Indices	Before treatment (*n* = 17)	Placebo (*n* = 17)	Lingbao Huxin Dan (*n* = 17)
SDNN (ms, M ± SD)	85.57 ± 40.25^a,b^	110.62 ± 45.46^a^	115.98 ± 35.16^b^
SDANN (ms, M ± SD)	74.05 ± 22.79^b^	89.65 ± 45.11	100.95 ± 35.05^b^
rMSSD (ms, M ± SD)	32.75 ± 21.72^b^	44.44 ± 40.98^c^	48.37 ± 24.46^b,c^
SDNN index (ms, M ± SD)	41.29 ± 24.97^b^	45.91 ± 15.79^c^	54.19 ± 34.09^b,c^
pNN50 (%, p25, p75)	5.21 (0.935, 8.830)	6.99 (1.26, 9.39)	4.67 (1.23, 9.46)

Propensity score matching was performed with a tolerance threshold (caliper width) of 0.05. Eight subjects were assigned a match status of 0, indicating failure to match and necessitating their exclusion from subsequent analyses. Superscript letters denote statistically significant differences: a, compared with placebo group before intervention; b, compared with Lingbao Huxin Dan group before intervention; c, between placebo and Lingbao Huxin Dan groups.

### 3.4 DCG findings

**Figure 4:**
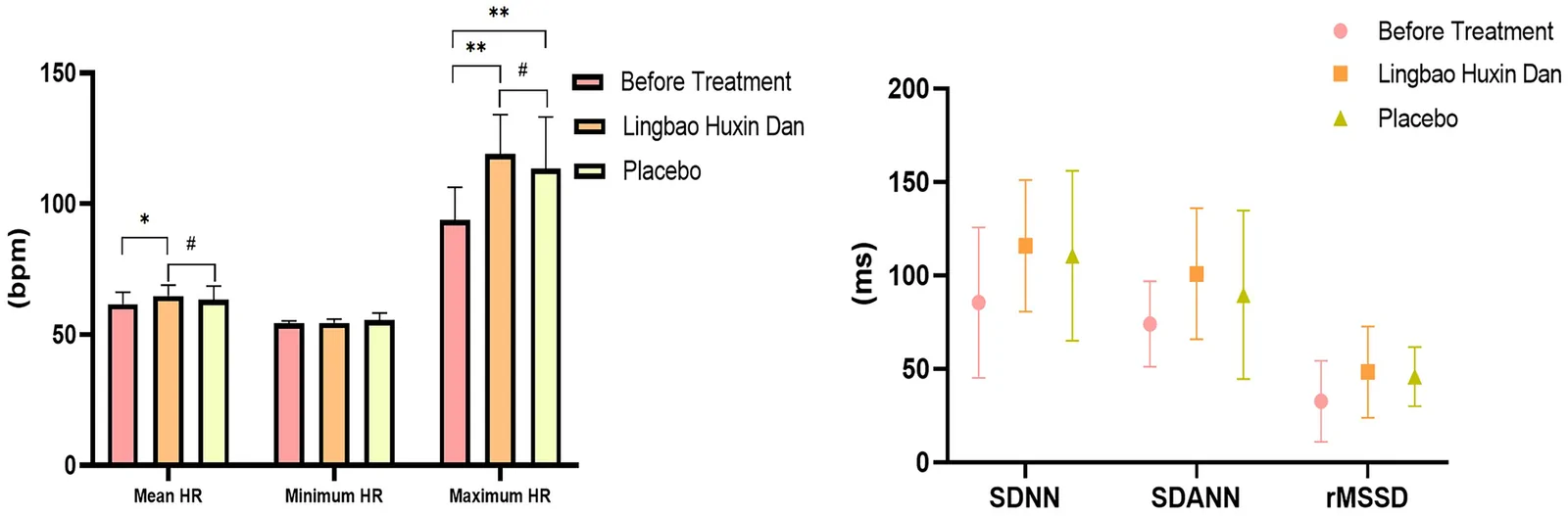
Changes in heart rate and time-domain indices of heart rate variability before and after intervention (bpm). Compared with the pre-intervention baseline, *P < 0.05 and **P < 0.01; compared with the placebo period, #*P* < 0.05. HR, heart rate.

Following intervention, there were observable increases in total heartbeat count, mean HR, minimum HR, and maximum HR. The increase in mean HR and peak HR was significantly more pronounced during the LBHXD phase than during placebo (*P* < 0.05). Specifically, mean HR increased from 61.65 ± 4.60 bpm to 64.76 ± 4.13 bpm, and maximum HR rose from 93.71 ± 12.58 bpm to 118.94 ± 15.14 bpm during active treatment (both *P* < 0.05), indicating improved chronotropic competence. Concurrently, the 24-h AP proportion derived from DCG monitoring significantly declined post-intervention, with a greater reduction observed during the LBHXD phase (Table 5; Figure 4), consistent with the pacemaker telemetry data.

**Table 5:** Changes in dynamic electrocardiographic parameters before and after each intervention period.

Parameters	Before treatment (*n* = 17)	Placebo (*n* = 17)	Lingbao Huxin Dan (*n* = 17)
Atrial Pacing Ratio (%, M ± SD)	72.50 ± 20.36^a,b^	52.64 ± 24.16^a^	46.65 ± 19.30^b^
Total Heartbeats (bpm, M ± SD)	84,822.76 ± 7,067.70^b^	84,587.80 ± 6,954.34^c^	87,179.64 ± 6,516.96^b,c^
Average Heart Rate (bpm, M ± SD)	61.65 ± 4.60^b^	63.35 ± 5.16^c^	64.76 ± 4.13^b,c^
Slowest Heart Rate (bpm, M ± SD)	54.18 ± 1.03	55.47 ± 2.77	54.47 ± 1.45
Fastest Heart Rate (bpm, M ± SD)	93.71 ± 12.58^a,b^	113.29 ± 19.85^a,c^	118.94 ± 15.14^b,c^
Duration of Bradycardia (h, p25, p75)	3.75 (0.96,19.13)	1.45 (0.46,15.03)	1.25 (0.50,13.43)

Propensity score matching was performed with a tolerance threshold (caliper width) of 0.05. Eight subjects were assigned a match status of 0, indicating failure to match and necessitating their exclusion from subsequent analyses. Superscript letters denote statistically significant differences: a, compared with placebo group before intervention; b, compared with Lingbao Huxin Dan group before intervention; c, between placebo and Lingbao Huxin Dan groups.

### 3.5 Echocardiographic outcomes

No significant changes were detected in left atrial anteroposterior diameter or LVEF following either intervention (*P* > 0.05). However, a numerically greater increase in LVEF was observed in the LBHXD phase compared to placebo, though this difference did not achieve statistical significance. This suggests a potential trend toward improved systolic function that may warrant further investigation in larger trials (Table 6).

**Table 6:** Changes in echocardiographic parameters before and after each intervention period.

Parameters	Before treatment (*n* = 17)	Placebo (*n* = 17)	Lingbao Huxin Dan (*n* = 17)
Left Atrial Anteroposterior Diameter (mm, M ± SD)	38.83 ± 4.71	38.67 ± 5.27	38.46 ± 5.54
Left Ventricular Ejection Fraction (%, M ± SD)	67.92 ± 4.21	68.78 ± 4.89	70.04 ± 5.06

Propensity score matching was performed with a tolerance threshold (caliper width) of 0.05. Eight subjects were assigned a match status of 0, indicating a failure to match and thus requiring their exclusion from subsequent analyses. No statistically significant differences were detected in any of the comparisons within the table (*P* > 0.05).

### 3.6 Safety assessment

The safety population included all participants who received at least one dose of the study product. In the control group, two participants reported non-serious adverse events during the LBHXD phase: one experienced palpitation and one reported transient limb numbness; both resolved spontaneously after drug cessation without medical intervention. One participant exhibited an isolated elevation in serum creatinine during the placebo phase, which normalized without treatment. In the experimental group, one participant developed paroxysmal atrial fibrillation, which was managed per standard clinical guidelines. No serious or unexpected adverse events related to the study medication were reported throughout the trial, indicating a favorable safety profile for LBHXD under the conditions tested.

## 4 Discussion

The present study demonstrates that LBHXD significantly reduces AP burden, as evidenced by both pacemaker-derived telemetry and DCG, while concurrently improving HRV and elevating key HR parameters in patients with SSS. Collectively, these objective findings reduced atrial pacing dependency, enhanced HRV, and increased heart rate suggest that LBHXD may exert beneficial effects on intrinsic sinoatrial node function and autonomic regulation, thereby potentially reducing reliance on atrial pacing. However, it should be noted that these mechanistic interpretations are speculative and require direct confirmation in future studies.

Beyond statistical significance, the clinical meaningfulness of the observed approximately 9% absolute reduction in atrial pacing percentage, warrants careful consideration from multiple perspectives. First, regarding pacemaker burden and battery longevity, a prospective, multicenter international study involving 302 patients demonstrated that pacing percentage is significantly correlated with both atrial and ventricular pacemaker remaining longevity (14). The CAPTURE Trial further reported that each percentage point reduction in pacing burden translates into measurable energy savings over the device lifetime, with algorithms for reducing atrial pacing associated with an estimated 0.2-year extension in projected longevity (15). Although this per-patient extension may appear modest, at the population level, given the hundreds of thousands of pacemakers implanted annually, it translates into substantial reductions in device replacement procedures, associated healthcare costs, and procedure-related complications. The AP reduction observed in our study suggests that LBHXD may offer a complementary pharmacological strategy for reducing pacing burden. Second, regarding clinical outcomes, a clinical study defined high AP burden as >50% and demonstrated its association with worse clinical outcomes, including reduced left ventricular reverse remodeling, increased risk of new-onset or recurrent atrial fibrillation, and higher rates of heart failure rehospitalization (16). Although the recent DANPACE II randomized trial suggested that atrial pacing minimization alone did not reduce the incidence of atrial fibrillation (17), the unique value of our study lies in its demonstration that pharmacological intervention with LBHXD may reduce atrial pacing through mechanisms such as autonomic nervous system modulation, while simultaneously maintaining necessary chronotropic support, a strategy fundamentally different from simply lowering the pacemaker base rate. Third, regarding symptom improvement, the reduction in AP in our study was accompanied by significant increases in both mean heart rate and maximum heart rate, as well as significant improvements in HRV parameters. These physiological improvements reflecting enhanced chronotropic competence and autonomic balance are mechanistically linked to symptom amelioration, including reduced dizziness, fatigue, and exercise intolerance, which are hallmark complaints of SSS patients. The improved intrinsic rhythm preservation suggested by the reduced AP indicates that LBHXD may enhance the patient's own sinoatrial node function, thereby reducing dependence on artificial pacing. Fourth, regarding the magnitude of effect, the 9.04% absolute reduction in AP observed in our study compares favorably with other pharmacological interventions for bradyarrhythmias. For example, theophylline is one of the few pharmacological agents with positive chronotropic effects in sinus node dysfunction; however, its effect is characterized by considerable variability and limited efficacy, with a lack of robust evidence for long-term benefit (18). Our findings suggest that LBHXD may offer a more consistent and clinically meaningful improvement in intrinsic cardiac rhythm. Collectively, these considerations support the clinical relevance of the observed AP reduction and underscore the potential role of LBHXD as a complementary therapeutic option for SSS patients with implanted pacemakers.

Current therapeutic options for SSS remain largely limited to permanent pacemaker implantation (19), which, although effective in preventing bradycardia-related symptoms and improving quality of life, does not address the underlying pathophysiology. In the United States alone, over 200,000 pacemakers are implanted annually (20). However, device-related complications—including lead dislodgement, pocket infection, venous thrombosis, and pneumothorax—occur in approximately 10% of cases (21). While leadless pacemakers eliminate transvenous leads and generator pockets, their clinical benefits in terms of long-term outcomes or functional improvement remain unproven (22). Emerging biological pacing strategies, such as stem cell or gene-based approaches to generate pacemaker-like cardiomyocytes, hold theoretical promise but face substantial translational barriers before clinical application (23). Therefore, there is a compelling need for safe and effective adjunctive therapies that can augment endogenous cardiac rhythm and reduce pacing dependency.

TCM has historically played a significant role in managing bradyarrhythmias, with documented use spanning more than two millennia. Rooted in the principles of holistic regulation and syndrome differentiation, TCM aims to restore physiological balance through modulation of Qi, blood, Yin, and Yang (24). A growing body of evidence supports the efficacy of several TCM formulations—such as Shenxian Shengmai Oral Liquid and Shensong Yangxin Capsule—in improving sinus node function and alleviating symptomatic bradycardia (25). Clinical reports suggest that TCM interventions may achieve efficacy rates of up to 65.22% within four weeks, with certain herbal components directly enhancing sinoatrial node automaticity (26). LBHXD, a multi-herb formulation, has been shown in prior mechanistic studies to activate the β-adrenergic receptor-mediated CAMP/PKA signaling pathway, thereby shortening atrioventricular conduction time and improving sinoatrial impulse generation. Additionally, it attenuates bradycardia-induced myocardial injury by promoting superoxide dismutase (SOD) activity and suppressing lactate dehydrogenase (LDH) elevation, underscoring its pharmacological potential in rhythm stabilization (11).

In this study, the concordant reduction in AP proportion—observed via both device interrogation and DCG—suggests a consistent enhancement of intrinsic sinus rhythm across different temporal scales: long-term (device-recorded trends) and short-term (daily rhythm dynamics). This dual-source consistency strengthens the validity of the observed effect. Data mining analyses have identified danshen and red ginseng as core herbs in TCM prescriptions for bradyarrhythmias (27). Notably, Tongyang Huoxue Decoction, which prominently features red ginseng, has demonstrated protective effects on sinoatrial node cells via mitochondrial pathway modulation (28). These prior findings, together with the results of the present study, raise the possibility that similar pathways may be involved in the effects of LBHXD, although this hypothesis requires direct testing in future mechanistic studies.

From a TCM perspective, “heart Qi” governs cardiac contractility and rhythmicity, while “blood” ensures adequate perfusion. The interplay between sufficient Qi, abundant blood, and unobstructed meridians forms the foundation of cardiovascular homeostasis (29). LBHXD's therapeutic strategy centers on tonifying heart Qi and promoting blood circulation. Red ginseng, a principal herb in the formula, is renowned for its ability to invigorate Qi and enhance myocardial energy metabolism, which may explain the observed increases in average, minimum, and maximum HR. These improvements likely contribute to better systemic perfusion and reduced risk of adverse cardiac remodeling associated with chronic bradycardia.

HRV is a well-established non-invasive index for assessing autonomic nervous system (ANS) function. By quantifying beat-to-beat variations in RR intervals, HRV provides critical insights into cardiovascular regulation and has been widely validated as a prognostic marker in both cardiovascular and chronic non-communicable diseases (30). The ANS is the primary regulator of HRV, with increased values typically reflecting enhanced parasympathetic (vagal) modulation and greater cardiovascular adaptability (31). Epidemiological evidence indicates that each 1-ms increase in SDNN is associated with approximately a 1% reduction in cardiovascular mortality risk (32). In this study, LBHXD intervention led to significant increases in both rMSSD and SDNN, suggesting improved autonomic balance. These changes imply attenuation of excessive vagal suppression—a common feature in SSS—and may contribute to enhanced sinoatrial node responsiveness. Furthermore, Danshen, a key component of LBHXD, possesses established blood-activating and stasis-resolving properties. Modern pharmacological studies have demonstrated its ability to improve myocardial microcirculation and reduce oxidative stress (29), which may synergistically optimize the cellular metabolic environment and support myocardial oxygenation. These mechanisms may constitute an important structural and functional foundation underlying the observed improvement in HRV.

It should be noted that pacemaker syndrome is primarily associated with AV dyssynchrony in single-chamber pacing and is less relevant to DDD pacing. All patients in our study had DDD pacemakers, which maintain AV synchrony and therefore minimize the risk of pacemaker syndrome. The observed benefits of LBHXD, reduced AP%, improved HRV, and increased heart rate, should be interpreted in the context of enhanced intrinsic sinus node function and autonomic regulation, rather than as a strategy to prevent pacemaker syndrome. The potential benefits of reducing AP% in DDD-paced patients relate to battery longevity, preservation of intrinsic atrioventricular synchrony, and potential reduction in long-term atrial remodeling, rather than prevention of pacemaker syndrome.

In summary, in patients with SSS following pacemaker implantation, the observed improvements in pacing burden, HRV, and heart rate parameters are consistent with the hypothesis that LBHXD may exert beneficial effects through multiple putative pathways, potentially including modulation of autonomic tone, enhancement of intrinsic sinoatrial node activity, and improvement in overall HR stability. However, it must be emphasized that these proposed mechanisms are speculative at this stage and are primarily derived from the observed physiological changes in conjunction with prior preclinical evidence, rather than from direct mechanistic measurements in the present study. These physiological improvements may reduce the long-term risk of arrhythmia-related complications such as atrial fibrillation and heart failure, thereby contributing to better clinical outcomes. The favorable safety profile observed supports its potential as a complementary therapeutic option, highlighting the value of integrating TCM with conventional cardiovascular care in rhythm management. It should be noted that cardiac autonomic dysfunction is not exclusive to cardiovascular conditions. Evidence suggests that patients with chronic obstructive pulmonary disease comorbid with obstructive sleep apnea frequently exhibit impaired autonomic regulation (33). Whether LBHXD can ameliorate autonomic imbalance in such non-cardiac contexts remains unknown and warrants dedicated investigation.

Despite these encouraging findings, several limitations warrant careful consideration. First, the limited sample size is the principal constraint of this pilot study. Although the randomized crossover design improves statistical efficiency by leveraging within-subject comparisons to control for inter-individual variability, the modest number of participants, especially following propensity score matching, likely attenuated statistical power to detect subtle yet clinically meaningful changes. Notably, this trial was explicitly designed as a proof-of-concept study to generate preliminary evidence on the efficacy and safety of LBHXD in patients with SSS who have received permanent pacemaker implantation, a population for which no prior randomized controlled trial data are available. The consistent positive signals observed across multiple endpoints thus provide both a robust rationale and empirically informed effect size estimates to inform the sample size calculation and design of future adequately powered, multicenter confirmatory trials. Second, the single-center design may introduce selection bias and affect the generalizability of the results. Third, the single-blind design constitutes a methodological limitation that warrants explicit acknowledgment. Although we implemented multiple prespecified bias-mitigation strategies, including centralized allocation concealment, identical packaging of active drug and placebo, protocol-specified standardized assessments, and blinded statistical analysis performed by independent researchers, investigator unblinding remains an inherent constraint. Notably, however, our primary endpoint and key secondary endpoints were predominantly objective, device-derived metrics, which are inherently less vulnerable to investigator influence than subjective, symptom-based assessments. That said, we acknowledge that a double-blind design would further enhance methodological rigor and eliminate residual performance and detection bias, particularly when patient-reported outcomes or symptom-driven endpoints are incorporated. This limitation has been transparently disclosed, and we recommend that future confirmatory trials adopt a double-blind framework to maximize internal validity. Fourth, we acknowledge that the multi-component profile of LBHXD introduces theoretical risks of residual bioactivity beyond the 2-week washout period, particularly for constituents with prolonged elimination half-lives or delayed clearance kinetics. While published pharmacokinetic studies demonstrate that most active moieties, including ginsenosides, notoginsenosides, salvianolic acids, and tanshinones, exhibit relatively short elimination half-lives (typically ranging from several hours to 48 h), inter-individual variability in oral bioavailability and pharmacokinetic disposition cannot be discounted. Additionally, the potential for active metabolite formation or enterohepatic recirculation, which may extend the biological activity of parent compounds, cannot be fully excluded. Accordingly, we recognize that the 2-week washout period may be suboptimal for mitigating carryover effects associated with long-acting components, and we explicitly identify this as a key limitation of the current study. To address this concern rigorously in future investigations, we recommend extending the washout period to 3-4 weeks, a duration aligned with methodological guidance for herbal formulations exhibiting complex pharmacokinetic profiles, to ensure complete elimination of residual drug-related effects. Fifth, we acknowledge that the absence of validated patient-reported outcome (PRO) instruments, specifically standardized symptom assessment scales and quality-of-life questionnaires, constitutes a key methodological limitation of the present study. SSS is inherently a symptom-driven disorder, defined by core clinical manifestations including dizziness, fatigue, palpitations, and reduced exercise tolerance; accordingly, therapeutic efficacy must be evaluated not only through objective physiological parameters but, critically, by meaningful improvements in patients' subjective symptom burden and functional capacity. While this study rigorously assessed objective physiological endpoints, these surrogate markers inherently lack sensitivity to the multidimensional nature of health-related quality of life and patient-centered symptom experience. Although patient-reported symptoms were systematically collected at each study visit as part of routine clinical monitoring, these qualitative reports were neither standardized nor quantified using psychometrically validated PRO tools. To strengthen the evidentiary basis for LBHXD in future research, we recommend that confirmatory multicenter trials prospectively integrate well-established, regulatory-endorsed PRO instruments, such as the 36-Item Short Form Health Survey (SF-36) and the EuroQol Five-Dimension Five-Level Questionnaire (EQ-5D-5L), as co-primary or prespecified key secondary endpoints. This approach will enable a robust, patient-centric evaluation of LBHXD's clinical impact and generate data suitable for regulatory review, health technology assessment, and shared decision-making between clinicians and patients. Although intermediate endpoints showed improvement, long-term clinical outcomes—including arrhythmia burden, hospitalization rates, and progression to permanent pacing—require confirmation in larger, multicenter trials with extended follow-up periods. Future studies should aim to expand the observation window to fully evaluate the longitudinal impact of LBHXD on SSS progression. Moreover, the precise bioactive constituents and their network pharmacodynamic interactions within this complex herbal formulation remain to be systematically characterized.

## 5 Conclusion

In conclusion, this randomized, single-blind, placebo-controlled crossover trial provides initial clinical evidence that Lingbao Huxin Dan, as an adjunctive therapy, significantly reduces atrial pacing dependency and improves key physiological parameters, including HRV and heart rate, in patients with sick sinus syndrome following pacemaker implantation. The consistency of these results across both device-based telemetry and 24- h Holter monitoring strengthens the validity of these observations. While the observed improvements in HRV and heart rate are consistent with the hypothesis that LBHXD may positively modulate autonomic function and sinoatrial node activity, the present study was not designed to provide direct mechanistic evidence for these interpretations, and such mechanisms remain speculative pending confirmatory studies. The intervention was well tolerated, with a favorable safety profile, supporting its acceptability in this patient population. These findings suggest that integrating this evidence-based traditional Chinese medicine formulation with standard device therapy may represent a promising integrative approach to the comprehensive management of SSS, potentially reducing long-term pacing burden. Future multicenter studies with larger sample sizes and extended follow-up periods are warranted to confirm these preliminary results, clarify the pharmacodynamic mechanisms underlying its multi-component, multi-target effects, and evaluate its impact on clinically meaningful endpoints.

## Data Availability

The raw data supporting the conclusions of this article will be made available by the authors, without undue reservation.
