# Supplementary material for: Efficacy and safety of Lingbao Huxin Dan in the treatment of sick sinus syndrome: a randomized, single-blind, placebo-controlled, crossover trial
Source: Front Cardiovasc Med. 2026 Aug 12;13:1821435. doi: 10.3389/fcvm.2026.1821435 (PMC13506683; doi:10.3389/fcvm.2026.1821435)
Supplement: Supplementary file 1 [file Table1.docx]

Supplementary Material

Supplementary Table 1. A total of 222 compounds identified from LBHXD by UPLC-Q-TOF-MS

| **NO.** | **RT（min）** | **m/z** | **Metabolite name** | **Formula** | **LBHXD** | **Adduct** |
| --- | --- | --- | --- | --- | --- | --- |
| 1 | 9.772417 | 297.1446 | Cryptotanshinone | C19H20O3 | 21789579264 | [M+H]+ |
| 2 | 8.603833 | 357.2739 | Arachidic acid | C20H40O2 | 21379913728 | [M-H+2Na]+ |
| 3 | 10.09622 | 131.0475 | Cinnamic acid | C9H8O2 | 17478569984 | [M-H2O+H]+ |
| 4 | 7.2579 | 417.2226 | Schizandrin A | C24H32O6 | 16048703488 | [M+H]+ |
| 5 | 11.37245 | 282.2755 | Oleamide | C18H35NO | 14034452480 | [M+H]+ |
| 6 | 6.082983 | 173.0772 | Suberic acid | C8H14O4 | 9310038016 | [M-H]- |
| 7 | 10.2803 | 295.1288 | Tanshinone IIA | C19H18O3 | 8946461696 | [M+H]+ |
| 8 | 0.9434834 | 191.0148 | Citric acid | C6H8O7 | 7818258944 | [M-H]- |
| 9 | 8.46315 | 387.2489 | Bufalin | C24H34O4 | 6135296000 | [M+H]+ |
| 10 | 8.8286 | 385.2327 | Resibufogenin | C24H32O4 | 4935241216 | [M+H]+ |
| 11 | 9.2235 | 311.1625 | Triptophenolide | C20H24O3 | 4583371264 | [M-H]- |
| 12 | 5.108883 | 127.0377 | Maltol | C6H6O3 | 4435788288 | [M+H]+ |
| 13 | 7.867116 | 403.2436 | Telocinobufagin | C24H34O5 | 4270784000 | [M+H]+ |
| 14 | 6.173683 | 135.0409 | 4-Hydroxyacetophenone | C8H8O2 | 3947319040 | [M-H]- |
| 15 | 8.603833 | 133.0633 | 3,4-Dimethylbenzoic acid | C9H10O2 | 3566677248 | [M+H-H2O]+ |
| 16 | 0.8166167 | 124.0034 | Taurine | C2H7NO3S | 3501104896 | [M-H]- |
| 17 | 9.898883 | 205.1929 | Nerolidol | C15H26O | 3476081152 | [M+H-H2O]+ |
| 18 | 9.7019 | 301.2123 | gamma-linolenic acid | C18H30O2 | 2636475136 | [M+Na]+ |
| 19 | 9.997316 | 511.3355 | Alisol F | C30H48O5 | 2242553600 | [M+Na]+ |
| 20 | 9.5754 | 317.2067 | Cafestol | C20H28O3 | 2203447296 | [M+H]+ |
| 21 | 5.916983 | 121.0255 | 4-Hydroxybenzaldehyde | C7H6O2 | 1997304704 | [M-H]- |
| 22 | 7.45665 | 425.3731 | Betulin | C30H50O2 | 1780169344 | [M+H-H2O]+ |
| 23 | 8.716217 | 391.2792 | Cholic acid | C24H40O5 | 1745357568 | [M-H2O+H]+ |
| 24 | 2.014883 | 188.0513 | Pyroglutamic acid | C5H7NO3 | 1619417600 | [M+CH3COO]- |
| 25 | 8.603833 | 118.064 | Indole | C8H7N | 1548117376 | [M+H]+ |
| 26 | 10.73285 | 303.2281 | Abietic acid | C20H30O2 | 1295670144 | [M+H]+ |
| 27 | 6.742983 | 441.3676 | Soyasapogenol M1 | C30H48O2 | 1252186240 | [M+H]+ |
| 28 | 8.673984 | 309.1084 | Loureirin A | C17H18O4 | 1176723968 | [M+Na]+ |
| 29 | 8.0084 | 457.2161 | Clerodin | C24H34O7 | 1172612224 | [M+Na]+ |
| 30 | 9.56115 | 279.2279 | Coronaric acid | C18H32O3 | 1140247424 | [M+H-H2O]+ |
| 31 | 8.84265 | 415.207 | Niranthin | C24H32O7 | 1091103104 | [M-H2O+H]+ |
| 32 | 7.562883 | 339.1287 | 6-Prenylnaringenin | C20H20O5 | 1076669568 | [M-H]- |
| 33 | 11.24255 | 473.3394 | Vitamin K1 | C31H46O2 | 1053633472 | [M+Na]+ |
| 34 | 5.406167 | 137.0199 | 3-Hydroxybenzoate | C7H6O3 | 1027776960 | [M-H]- |
| 35 | 3.558133 | 268.1002 | Adenosine | C10H13N5O4 | 984592256 | [M+H]+ |
| 36 | 10.8884 | 263.2333 | Linoleic acid | C18H32O2 | 974754816 | [M-H2O+H]+ |
| 37 | 9.2226 | 279.0985 | 1,2-Dihydrotanshinquinone | C18H14O3 | 916219264 | [M+H]+ |
| 38 | 9.74405 | 277.0826 | Tanshinone | C18H12O3 | 813837632 | [M+H]+ |
| 39 | 9.4199 | 315.1919 | 7-Oxodehydroabietic acid | C20H26O3 | 807978432 | [M+H]+ |
| 40 | 8.881733 | 297.1466 | Auraptene | C19H22O3 | 779007488 | [M-H]- |
| 41 | 7.598017 | 163.0368 | Umbelliferone | C9H6O3 | 763286464 | [M+H]+ |
| 42 | 9.800433 | 105.069 | Styrene | C8H8 | 747975616 | [M+H]+ |
| 43 | 6.606616 | 147.0407 | p-Coumaraldehyde | C9H8O2 | 747885952 | [M-H]- |
| 44 | 6.203133 | 163.0351 | p-Coumaric acid | C9H8O3 | 692257344 | [M-H]- |
| 45 | 8.673984 | 308.1236 | Piperine | C17H19NO3 | 687860544 | [M+Na]+ |
| 46 | 6.592317 | 195.0645 | Asaronaldehyde | C10H12O4 | 645539904 | [M-H]- |
| 47 | 1.891433 | 117.0153 | Succinic acid | C4H6O4 | 633322112 | [M-H]- |
| 48 | 9.279083 | 205.1923 | Farnesol | C15H26O | 600600960 | [M+H-H2O]+ |
| 49 | 9.871017 | 277.213 | Stearidonic acid | C18H28O2 | 571708224 | [M+H]+ |
| 50 | 9.5086 | 293.173 | Embelin | C17H26O4 | 564529728 | [M-H]- |
| 51 | 7.3893 | 165.0508 | 4-O-Methylvanillin | C9H10O3 | 556740160 | [M-H]- |
| 52 | 0.9295334 | 173.0043 | cis-Aconitate | C6H6O6 | 544659456 | [M-H]- |
| 53 | 9.335317 | 149.0944 | cis-Anethole | C10H12O | 489450752 | [M+H]+ |
| 54 | 3.825617 | 134.0429 | Adenine | C5H5N5 | 473121088 | [M-H]- |
| 55 | 7.315166 | 144.0791 | 1-Naphthylamine | C10H9N | 453773088 | [M+H]+ |
| 56 | 8.491283 | 95.04863 | Sorbic acid | C6H8O2 | 434983744 | [M-H2O+H]+ |
| 57 | 8.84265 | 194.1153 | Salsoline | C11H15NO2 | 430694528 | [M+H]+ |
| 58 | 11.05682 | 503.3407 | Madecassic acid | C30H48O6 | 429880832 | [M-H]- |
| 59 | 9.772417 | 425.3358 | delta-tocopherol | C27H46O2 | 410980704 | [M+Na]+ |
| 60 | 11.24255 | 265.2491 | Oleic acid | C18H34O2 | 408192224 | [M+H-H2O]+ |
| 61 | 8.435066 | 153.1254 | Camphor | C10H16O | 372790464 | [M+H]+ |
| 62 | 7.300567 | 352.1493 | Palmatine | C21H22NO4 | 367864640 | [M]+ |
| 63 | 10.46417 | 201.1612 | Nootkatone | C15H22O | 367122912 | [M+H-H2O]+ |
| 64 | 10.0822 | 383.1953 | Corynoxeine | C22H26N2O4 | 363513344 | [M+H]+ |
| 65 | 9.898883 | 219.1713 | alpha-Cyperone | C15H22O | 354959776 | [M+H]+ |
| 66 | 7.923683 | 167.0682 | Paeonol | C9H10O3 | 352156672 | [M+H]+ |
| 67 | 0.9575167 | 85.026 | Crotonic acid | C4H6O2 | 345483584 | [M-H]- |
| 68 | 7.055383 | 354.1289 | Protopine | C20H19NO5 | 338952512 | [M+H]+ |
| 69 | 1.948017 | 257.1214 | Pterostilbene | C16H16O3 | 325376416 | [M+H]+ |
| 70 | 1.948017 | 331.1202 | Toddalolactone | C16H20O6 | 310198592 | [M+Na]+ |
| 71 | 5.010716 | 190.0842 | Synephrine | C9H13NO2 | 303512544 | [M+Na]+ |
| 72 | 9.109783 | 107.0481 | 3-Hydroxybenzyl alcohol | C7H8O2 | 298922080 | [M+H-H2O]+ |
| 73 | 8.505383 | 99.04322 | alpha-Methylene butyrolactone | C5H6O2 | 284407776 | [M+H]+ |
| 74 | 7.399917 | 139.0373 | 4-Hydroxybenzoic acid | C7H6O3 | 278590336 | [M+H]+ |
| 75 | 10.03988 | 221.1872 | Caryophyllene Epoxide | C15H24O | 251891776 | [M+H]+ |
| 76 | 9.64565 | 135.1153 | Carveol | C10H16O | 247458128 | [M+H-H2O]+ |
| 77 | 10.26622 | 299.16 | 6-Shogaol | C17H24O3 | 247457728 | [M+Na]+ |
| 78 | 9.363483 | 416.3105 | Glycolithocholic acid | C26H43NO4 | 246243248 | [M-H2O+H]+ |
| 79 | 5.43605 | 286.1039 | Arborinine | C16H15NO4 | 242736016 | [M+H]+ |
| 80 | 7.583917 | 439.3518 | Oleanolic acid | C30H48O3 | 240148016 | [M+H-H2O]+ |
| 81 | 7.214967 | 285.0715 | Calycosin | C16H12O5 | 238339712 | [M+H]+ |
| 82 | 8.46315 | 163.0734 | Methyl cinnamate | C10H10O2 | 235103488 | [M+H]+ |
| 83 | 9.462116 | 269.1137 | Arglabin | C15H18O3 | 234327344 | [M+Na]+ |
| 84 | 0.8477 | 175.1165 | Arginine | C6H14N4O2 | 230977888 | [M+H]+ |
| 85 | 0.9575167 | 89.02093 | Lactic acid | C3H6O3 | 230143520 | [M-H]- |
| 86 | 9.631667 | 267.1742 | Boldione | C19H24O2 | 229628784 | [M+H-H2O]+ |
| 87 | 8.491283 | 125.0583 | Orcinol | C7H8O2 | 220966448 | [M+H]+ |
| 88 | 9.0395 | 313.1398 | Eriosematin | C19H20O4 | 220492944 | [M+H]+ |
| 89 | 9.1803 | 280.1662 | Schinifoline | C17H23NO | 206686672 | [M+Na]+ |
| 90 | 8.353416 | 133.0099 | Malic acid | C4H4O5-2 | 202024384 | [M-H]- |
| 91 | 8.435066 | 311.1236 | Zaluzanin D | C17H20O4 | 191572432 | [M+Na]+ |
| 92 | 9.772417 | 177.1616 | Dihydro-alpha-ionone | C13H22O | 187061104 | [M+H-H2O]+ |
| 93 | 11.11497 | 417.3398 | Smilagenin | C27H44O3 | 185084944 | [M+H]+ |
| 94 | 9.56115 | 137.1308 | alpha-Pinene | C10H16 | 184269968 | [M+H]+ |
| 95 | 7.17235 | 320.0875 | Coptisine | C19H14NO4+ | 179547440 | [M]+ |
| 96 | 9.64565 | 161.0576 | 4-Methoxycinnamic acid | C10H10O3 | 175385808 | [M+H-H2O]+ |
| 97 | 1.5903 | 123.054 | Nicotinamide | C6H6N2O | 172456560 | [M+H]+ |
| 98 | 4.86245 | 176.0684 | Indole-3-acetic acid | C10H9NO2 | 167732016 | [M+H]+ |
| 99 | 8.349517 | 259.1667 | Curdione | C15H24O2 | 161124544 | [M+Na]+ |
| 100 | 5.71635 | 166.0843 | Phenylalanine | C9H11NO2 | 160170848 | [M+H]+ |
| 101 | 4.957233 | 167.0303 | 4-Methoxysalicylic acid | C8H8O4 | 159587376 | [M-H]- |
| 102 | 0.87745 | 365.1014 | Methylophiopogonanone A | C19H18O6 | 157543744 | [M+Na]+ |
| 103 | 8.378417 | 159.1149 | Myrcene | C10H16 | 153630352 | [M+Na]+ |
| 104 | 7.214967 | 133.0633 | Cinnamaldehyde | C9H8O | 151720096 | [M+H]+ |
| 105 | 5.76505 | 193.0458 | Ferulic acid | C10H10O4 | 151482848 | [M-H]- |
| 106 | 4.999967 | 94.02624 | 3-Pyridinol | C5H5NO | 145168128 | [M-H]- |
| 107 | 6.158683 | 179.03 | Caffeic acid | C9H8O4 | 134950016 | [M-H]- |
| 108 | 9.96935 | 377.1816 | Rauwolscine | C21H26N2O3 | 134282944 | [M+Na]+ |
| 109 | 5.511233 | 165.0145 | Terephthalic acid | C8H6O4 | 131072336 | [M-H]- |
| 110 | 4.898583 | 125.0201 | Pyrogallol | C6H6O3 | 129779992 | [M-H]- |
| 111 | 10.2381 | 477.3329 | Ursonic acid | C30H46O3 | 129280352 | [M+Na]+ |
| 112 | 8.050583 | 333.2017 | Andrographolide | C20H30O5 | 128613240 | [M+H-H2O]+ |
| 113 | 9.898883 | 443.2384 | Cinobufagin | C26H34O6 | 127756208 | [M+H]+ |
| 114 | 1.40565 | 124.0381 | Nicotinic acid | C6H5NO2 | 125332520 | [M+H]+ |
| 115 | 4.296783 | 267.0673 | Pratol | C16H12O4 | 122511616 | [M-H]- |
| 116 | 5.6745 | 151.0353 | (R)-mandelic Acid | C8H8O3 | 119192704 | [M-H]- |
| 117 | 8.898916 | 298.1393 | Stepharine | C18H19NO3 | 119122320 | [M+H]+ |
| 118 | 9.631667 | 271.1299 | Arteannuin B | C15H20O3 | 117698456 | [M+Na]+ |
| 119 | 4.746533 | 100.075 | Valine | C5H11NO2 | 114259224 | [M-H2O+H]+ |
| 120 | 5.108883 | 329.1783 | Crocetin | C20H24O4 | 112545968 | [M+H]+ |
| 121 | 13.48488 | 121.0254 | Benzoic acid | C7H6O2 | 108068704 | [M-H]- |
| 122 | 7.99445 | 310.1393 | Piperanine | C17H21NO3 | 107630560 | [M+Na]+ |
| 123 | 7.399917 | 167.0682 | 3-Phenyllactic acid | C9H10O3 | 106584416 | [M+H]+ |
| 124 | 1.472117 | 302.1305 | Dehydroevodiamine | C19H15N3O | 106249520 | [M+H]+ |
| 125 | 10.2381 | 378.1649 | Tetrahydropalmatine | C21H25NO4 | 103883104 | [M+Na]+ |
| 126 | 1.5903 | 275.132 | Gigantol | C16H18O4 | 103109224 | [M+H]+ |
| 127 | 5.403767 | 201.0997 | Harmalol | C12H12N2O | 98669112 | [M+H]+ |
| 128 | 4.898583 | 151.0354 | Methyl salicylate | C8H8O3 | 95668528 | [M-H]- |
| 129 | 0.9589167 | 146.0906 | gamma-Guanidinobutyric acid | C5H11N3O2 | 95188768 | [M+H]+ |
| 130 | 9.895066 | 337.1989 | Dodecyl gallate | C19H30O5 | 94883672 | [M-H]- |
| 131 | 2.4771 | 86.05959 | 2-Pyrrolidone | C4H7NO | 94129008 | [M+H]+ |
| 132 | 6.444917 | 163.0352 | m-Coumaric acid | C9H8O3 | 88603840 | [M-H]- |
| 133 | 6.049833 | 193.0474 | Scopoletin | C10H8O4 | 88117960 | [M+H]+ |
| 134 | 8.449067 | 295.0928 | 7-Acetoxy-2-Methylisoflavone | C18H14O4 | 87319776 | [M+H]+ |
| 135 | 7.980217 | 269.0772 | Formononetin | C16H12O4 | 85950168 | [M+H]+ |
| 136 | 8.054717 | 383.1995 | Isorhynchophylline | C22H28N2O4 | 82927992 | [M-H]- |
| 137 | 5.94705 | 167.0303 | Vanillic acid | C8H8O4 | 82676920 | [M-H]- |
| 138 | 4.192217 | 152.0548 | Guanine | C5H5N5O | 81767632 | [M+H]+ |
| 139 | 6.728267 | 370.1602 | Allocryptopine | C21H23NO5 | 78809832 | [M+H]+ |
| 140 | 0.9434834 | 357.0955 | Corymbosin | C19H18O7 | 78692712 | [M-H]- |
| 141 | 5.840766 | 191.0512 | Quinic acid | C7H12O6 | 76395744 | [M-H]- |
| 142 | 5.14115 | 267.142 | Thermopsine | C15H20N2O | 75845984 | [M+Na]+ |
| 143 | 5.872317 | 177.0144 | Daphnetin | C9H6O4 | 73874104 | [M-H]- |
| 144 | 7.867116 | 325.2151 | Kaurenic acid | C20H30O2 | 73868072 | [M+Na]+ |
| 145 | 7.541483 | 413.1901 | Prostratin | C22H30O6 | 73490432 | [M+Na]+ |
| 146 | 4.839633 | 153.0145 | 2,3-Dihydroxybenzoate | C7H6O4 | 70986336 | [M-H]- |
| 147 | 8.107233 | 381.2018 | Clausarin | C24H28O4 | 68997520 | [M+H]+ |
| 148 | 10.7894 | 395.1956 | Brucine | C23H26N2O4 | 68169680 | [M+H]+ |
| 149 | 0.8452666 | 177.0356 | Gulonic acid | C6H10O6 | 68004040 | [M-H]- |
| 150 | 4.780183 | 186.0889 | 3-Indolebutyric acid | C12H13NO2 | 64652304 | [M+H-H2O]+ |
| 151 | 6.757517 | 147.1149 | Lysine | C6H14N2O2 | 63792744 | [M+H]+ |
| 152 | 2.4329 | 151.0217 | Xanthine | C5H4N4O2 | 61996976 | [M-H]- |
| 153 | 9.81455 | 539.2949 | Ganoderic Acid A | C30H44O7 | 61638616 | [M+Na]+ |
| 154 | 4.898583 | 123.0409 | Salicyl alcohol | C7H8O2 | 59481320 | [M-H]- |
| 155 | 1.760617 | 182.079 | Tyrosine | C9H11NO3 | 56942624 | [M+H]+ |
| 156 | 5.999317 | 503.2641 | Neoandrographolide | C26H40O8 | 56570604 | [M+Na]+ |
| 157 | 5.482067 | 153.0149 | Gentisic acid | C7H6O4 | 55272048 | [M-H]- |
| 158 | 5.044517 | 109.0256 | Catechol | C6H6O2 | 54429528 | [M-H]- |
| 159 | 6.326317 | 181.0458 | Syringaldehyde | C9H10O4 | 53028172 | [M-H]- |
| 160 | 7.216767 | 283.0547 | Acacetin | C16H12O5 | 52828304 | [M-H]- |
| 161 | 13.66723 | 111.043 | Isobutyric acid | C4H8O2 | 52561400 | [M+Na]+ |
| 162 | 9.490334 | 527.3302 | Arjugenin | C30H48O6 | 49878760 | [M+Na]+ |
| 163 | 6.960166 | 322.1399 | Codeine | C18H21NO3 | 48748076 | [M+Na]+ |
| 164 | 4.679783 | 265.1262 | Huperzine A | C15H18N2O | 48021440 | [M+Na]+ |
| 165 | 0.8477 | 203.0497 | Psicose | C6H12O6 | 48004088 | [M+Na]+ |
| 166 | 5.982517 | 303.0467 | Quercetin | C15H10O7 | 47814644 | [M+H]+ |
| 167 | 4.696567 | 427.1765 | Anomalin | C24H26O7 | 47555416 | [M+H]+ |
| 168 | 8.0084 | 503.3312 | Brassinolide | C28H48O6 | 47551760 | [M+Na]+ |
| 169 | 5.452317 | 336.1621 | N6-Isopentenyladenosine | C15H21N5O4 | 46841144 | [M+H]+ |
| 170 | 6.201017 | 368.1091 | Bicuculline | C20H17NO6 | 46818196 | [M+H]+ |
| 171 | 5.255967 | 187.0844 | 2,3-Trimethylene-4-quinazolone | C11H10N2O | 46224920 | [M+H]+ |
| 172 | 5.666117 | 247.1413 | Hypaphorine | C14H18N2O2 | 45580236 | [M+H]+ |
| 173 | 6.049833 | 200.0683 | Dictamnine | C12H9NO2 | 42968040 | [M+H]+ |
| 174 | 6.679417 | 144.0408 | Indole-3-carboxyaldehyde | C9H7NO | 42450232 | [M-H]- |
| 175 | 6.728267 | 231.0257 | Fraxetin | C10H8O5 | 37670840 | [M+Na]+ |
| 176 | 0.9730167 | 311.0701 | Dianthoside | C12H16O8 | 37240104 | [M+Na]+ |
| 177 | 7.09955 | 433.2173 | Colforsin | C22H34O7 | 35610148 | [M+Na]+ |
| 178 | 1.5903 | 238.0677 | Kinetin | C10H9N5O | 34112296 | [M+Na]+ |
| 179 | 6.099933 | 110.0592 | 4-Aminophenol | C6H7NO | 33102836 | [M+H]+ |
| 180 | 4.696567 | 189.1 | Vasicine | C11H12N2O | 32288844 | [M+H]+ |
| 181 | 7.055383 | 169.0474 | Phloroacetophenone | C8H8O4 | 30641442 | [M+H]+ |
| 182 | 4.971317 | 114.9995 | Fumaric acid | C4H4O4 | 29936094 | [M-H]- |
| 183 | 2.2031 | 156.075 | Histidine | C6H9N3O2 | 28487706 | [M+H]+ |
| 184 | 7.329267 | 336.1183 | Berberine | C20H18NO4 | 27818096 | [M]+ |
| 185 | 0.8935 | 689.2006 | 2-Acetylacteoside | C31H38O16 | 25564002 | [M+Na]+ |
| 186 | 5.452317 | 140.069 | Betaine | C5H11NO2 | 25445240 | [M+Na]+ |
| 187 | 2.100783 | 113.0336 | Uracil | C4H4N2O2 | 25060816 | [M+H]+ |
| 188 | 5.733 | 357.2084 | Lathyrol | C20H30O4 | 22440522 | [M+Na]+ |
| 189 | 8.2074 | 645.4286 | Ginsenoside Rh2 | C36H62O8 | 22336580 | [M+Na]+ |
| 190 | 10.95925 | 256.2652 | Hexadecanamide | C16H33NO | 21138724 | [M+H]+ |
| 191 | 5.386766 | 455.2079 | Schizandrin | C24H32O7 | 20902402 | [M+Na]+ |
| 192 | 1.422133 | 273.1162 | Vestitol | C16H16O4 | 20788818 | [M+H]+ |
| 193 | 5.337533 | 439.2143 | Kurarinone | C26H30O6 | 19961978 | [M+H]+ |
| 194 | 5.666117 | 218.1149 | Securinine | C13H15NO2 | 19547668 | [M+H]+ |
| 195 | 5.484233 | 217.0949 | 1,2,3,4-Tetrahydro-beta-carboline-3-carboxylic acid | C12H12N2O2 | 19347404 | [M+H]+ |
| 196 | 4.3708 | 180.0997 | Salsolinol | C10H13NO2 | 19315308 | [M+H]+ |
| 197 | 6.3703 | 413.2339 | Tussilagone | C23H34O5 | 19296840 | [M+Na]+ |
| 198 | 5.566683 | 112.0382 | 2-Pyrrolecarboxylic acid | C5H5NO2 | 19185956 | [M+H]+ |
| 199 | 11.42962 | 459.3867 | Panaxatriol | C30H52O4 | 18941296 | [M+H-H2O]+ |
| 200 | 1.003867 | 455.1111 | Asperulosidic acid | C18H24O12 | 17589394 | [M+Na]+ |
| 201 | 5.010716 | 441.1921 | Gomisin H | C23H30O7 | 17180436 | [M+Na]+ |
| 202 | 5.468233 | 138.0536 | Trigonelline | C7H7NO2 | 16730064 | [M+H]+ |
| 203 | 6.83265 | 342.1651 | Isocorydine | C20H23NO4 | 16694006 | [M+H]+ |
| 204 | 5.0431 | 401.1987 | Rubschisandrin | C23H28O6 | 15080959 | [M+H]+ |
| 205 | 5.7991 | 169.074 | beta-carboline | C11H8N2 | 15007424 | [M+H]+ |
| 206 | 6.083017 | 177.0526 | Herniarin | C10H8O3 | 14458347 | [M+H]+ |
| 207 | 6.787667 | 532.2869 | Germine | C27H43NO8 | 13688851 | [M+Na]+ |
| 208 | 6.183683 | 287.0518 | Kaempferol | C15H10O6 | 12507060 | [M+H]+ |
| 209 | 5.43605 | 255.1094 | Melatonin | C13H16N2O2 | 12391465 | [M+Na]+ |
| 210 | 4.895916 | 330.2091 | Hetisine | C20H27NO3 | 11656058 | [M+H]+ |
| 211 | 5.403767 | 355.1929 | Dehydroandrographolide | C20H28O4 | 11261876 | [M+Na]+ |
| 212 | 4.812033 | 175.0822 | Indole-3-acetamide | C10H10N2O | 10916821 | [M+H]+ |
| 213 | 6.472367 | 235.0575 | Methyl syringate | C10H12O5 | 9397014 | [M+Na]+ |
| 214 | 1.3376 | 112.0495 | Cytosine | C4H5N3O | 9317163 | [M+H]+ |
| 215 | 3.898167 | 100.075 | Methylpyrrolidone | C5H9NO | 9086892 | [M+H]+ |
| 216 | 6.489067 | 317.0616 | 3-Methylquercetin | C16H12O7 | 8925699 | [M+H]+ |
| 217 | 4.199433 | 284.0959 | Guanosine | C10H13N5O5 | 6966299.5 | [M+H]+ |
| 218 | 2.032717 | 274.0885 | Cordycepin | C10H13N5O3 | 6085918 | [M+Na]+ |
| 219 | 6.62055 | 623.2951 | Ranaconitine | C32H44N2O9 | 4386559.5 | [M+Na]+ |
| 220 | 6.438933 | 745.4106 | Prosapogenin A | C39H62O12 | 4175399 | [M+Na]+ |
| 221 | 6.505816 | 789.436 | Ziyuglycoside I | C41H66O13 | 3323702.75 | [M+Na]+ |
| 222 | 4.400466 | 144.0459 | Hemineurine | C6H9NOS | 2845515.75 | [M+H]+ |
